# Development of a dual-flow tissue perfusion device for modeling the gastrointestinal tract–brain axis

**DOI:** 10.1063/5.0168953

**Published:** 2023-10-11

**Authors:** Lydia Baldwin, Emily J. Jones, Alexander Iles, Simon R. Carding, Nicole Pamme, Charlotte E. Dyer, John Greenman

**Affiliations:** 1 Centre of Biomedical Sciences, Hull York Medical School, University of Hull, Cottingham Road, Hull, HU6 7RX, United Kingdom; 2 Quadram Institute Bioscience, Rosalind Franklin Road, Norwich Research Park, Norwich, NR4 7UQ, United Kingdom; 3 Department of Materials and Environmental Chemistry, Stockholm University, 106 91 Stockholm, Sweden; 4 Norwich Medical School, University of East Anglia, Norwich Research Park, Norwich, NR4 7TJ, United Kingdom

## Abstract

Despite the large number of microfluidic devices that have been described over the past decade for the study of tissues and organs, few have become widely adopted. There are many reasons for this lack of adoption, primarily that devices are constructed for a single purpose or because they are highly complex and require relatively expensive investment in facilities and training. Here, we describe a microphysiological system (MPS) that is simple to use and provides fluid channels above and below cells, or tissue biopsies, maintained on a disposable, poly(methyl methacrylate), carrier held between polycarbonate outer plates. All other fittings are standard Luer sizes for ease of adoption. The carrier can be coated with cells on both sides to generate membrane barriers, and the devices can be established in series to allow medium to flow from one cell layer to another. Furthermore, the carrier containing cells can be easily removed after treatment on the device and the cells can be visualized or recovered for additional off-chip analysis. A 0.4 *μ*m membrane with cell monolayers proved most effective in maintaining separate fluid flows, allowing apical and basal surfaces to be perfused independently. A panel of different cell lines (Caco-2, HT29-MTX-E12, SH-SY5Y, and HUVEC) were successfully maintained in the MPS for up to 7 days, either alone or on devices connected in series. The presence of tight junctions and mucin was expressed as expected by Caco-2 and HT-29-MTX-E12, with Concanavalin A showing uniform staining. Addition of Annexin V and PI showed viability of these cells to be >80% at 7 days. Bacterial extracellular vesicles (BEVs) produced by *Bacteroides thetaiotaomicron* and labeled with 1,1′-dioctadecyl-3,3,3′,3′-tetramethylindocarbo-cyanine perchlorate (DiD) were used as a model component of the human colonic microbiota and were visualized translocating from an apical surface containing Caco-2 cells to differentiated SH-SY5Y neuronal cells cultured on the basal surface of connected devices. The newly described MPS can be easily adapted, by changing the carrier to maintain spheroids, pieces, or slices of biopsy tissue and joined in series to study a variety of cell and tissue processes. The cell layers can be made more complex through the addition of multiple cell types and/or different patterning of extracellular matrix and the ability to culture cells adjacent to one another to allow study of cell:cell transfer, e.g., passive or active drug transfer, virus or bacterial entry or BEV uptake and transfer.

## I. INTRODUCTION

Microfluidics provides an alternative to the traditional static cell line model due to the presence of flow, providing an important physiologically relevant component to the model. A number of devices incorporating multiple cell types and various support scaffolds have been described (Nolan *et al.*, 2023); however, these tend to be highly specialized for a specific tissue and/or function. Here, we describe a generic platform that can be modified to study monolayers and tissues, alone or joined in series. One of the most widely used microfluidic devices is the Colon Intestine-Chip™ produced by Emulate Inc. in which maintained epithelial cells display well-defined tight junctions and low permeability, as originally described by Kim *et al.* (2012), and then adapted to include co-culture with bacteria (Kim and Ingber, 2013). Although applicable to many established cell lines, studies with this chip have principally used Caco-2 which was originally derived from a colon adenocarcinoma and consistently forms cell monolayers on a semi-permeable membrane. Kim and colleagues also demonstrated that these chips reproduce the *in vivo* 3D structural and biomechanical features such as peristalsis (Kim *et al.*, 2012). More recently developed devices contain cell lines from different organs, e.g., colon (Caco-2) and liver (HepG2), separated by a porous membrane that allows metabolite transfer, with the aim of assessing drug metabolism (Choe *et al.*, 2017). Others have hypothesized that the *in vivo* gastrointestinal tract (GIT) environment is better reflected by co-culturing Caco-2 and HT-29 cell lines, with the latter producing mucin which is an important component of mucosa (Santbergen *et al.*, 2020). Alternatively, groups have focused on the importance of the extracellular matrix (ECM), to replicate the *in vivo* environment, with flow rates, gas concentration, and ECM components combining to affect cell behavior in 2D and 3D cultures in microfluidic devices; reviewed by Goy *et al.* (2019). The reality is that a true model of the complete GIT does not exist and there is, therefore, a need to ensure that any devices have their uses and limitations explicitly stated. Replicating brain pathophysiology on a microfluidic device has the added complication that it is essential to incorporate the blood brain barrier (BBB) as this is the interface between the peripheral blood system and the central nervous system. The BBB must be efficiently circumvented if drugs are to reach the neural tissue and treat diseases as diverse as Alzheimer's, Parkinson's or malignancies. As for the GIT chips, many different approaches have been followed to create devices, recently reviewed by Kawakita *et al.* (2022). A focus of many BBB devices has been the integration of a transepithelial electrical resistance (TEER) system to assess the integrity of the endothelial barrier of human brain microvascular endothelial cells (hBMECs), co-cultured with various other cells including astrocytes and pericytes. Palma-Florez *et al.* (2023) have recently developed a model, using human cell lines that measured the efficiency of peptide-targeting, polyethylene glycol functionalized gold nanoparticles. Alternatively, Vatine *et al.* (2019) used induced pluripotent stem cell (iPSC)-derived brain microvascular endothelial-like cells, astrocytes, and neurons to create a human BBB unit. Physiologically relevant TEER values were generated and the device showed protection of neural cells from plasma-induced toxicity. Despite the many benefits of the new devices both the GIT and BBB chips have limitations including complexity of manufacture, steep learning curves, limited device usability and flexibility and cost, i.e., can the device, or part of the platform, be re-used or are they entirely disposable?

It is clear that the GIT is central in health and many diseases, with its function and impact extending beyond a role in nutrient absorption. It is the largest immune organ in the body and accommodates 10^13^–10^14^ microorganisms that collectively make up the GIT microbiome which is integral to maintaining health (Seton and Carding, 2019). The gut-on-chip technology and interactions between tissues has been comprehensively reviewed by Ashammakhi *et al.* (2020) and Guo *et al.* (2023), highlighting the crosstalk between the gut-axis and other organs, in particular, the gut–liver and gut–brain interactions. In addition, the gut has been included in several body-on-a-chip approaches, in which multiple “organs” are established and interconnected, e.g., Maoz *et al.* (2018). Recently, there have been attempts to alter the GIT microbiome to improve gut health and/or ameliorate illness using a variety of microfluidic devices. For example, investigating the effects of oxygen concentration (Dickson, 2019), different diets (Garcia-Gutierrez and Cotter, 2021), and direct introduction of new microbial populations (Jalili-Firoozinezhad *et al.*, 2019). Although these models have demonstrated proof of concept, and the ability to modulate and measure effect, they have not yet been widely adopted, nor have they facilitated a reduction in the reliance on animal models. Obstacles to adoption include the complex nature of some of the devices, restricted access to cells or tissues, and/or assay problems including lack of robustness or sensitivity of detection (Sathish and Shen, 2021).

The current study describes a flexible and robust tissue perfusion platform that can be used to address many of the shortcomings of current devices. It comprises a monolayer of cultured adherent epithelial cells, juxtaposed to an endothelial cell line, mimicking the blood-tissue barrier. The purpose of using *Bacteroides thetaiotaomicron* (Bt) produced bacterial extracellular vesicles (BEVs) in this system is to determine if the microfluidics based organ-on-a-chip system we have developed can reproduce the biodistribution of BEVs *in vivo* that we have previously reported on (Jones *et al.*, 2020). BEVs are nano-size vesicles naturally produced by Gram-negative bacteria that mediate many microbe–microbe and microbe–host interactions (Juodeikis and Carding, 2022). We have previously shown that BEVs produced by Bt mediate interactions with host cells of the GIT and are trafficked via an intracellular (uptake) or paracellular (transmigration) route to cross the intestinal epithelium *in vivo* and reach distant tissues such as liver and brain (Jones *et al.*, 2020 and Modasia *et al.*, 2023). Here, Bt BEVs were used to visualize translocation between the semi-permeable organ compartments of the connected, dual-flow, perfusion devices.

## II. MATERIALS AND METHODS

### A. Design and fabrication of a dual-flow perfusion device

**FIG. 1.**
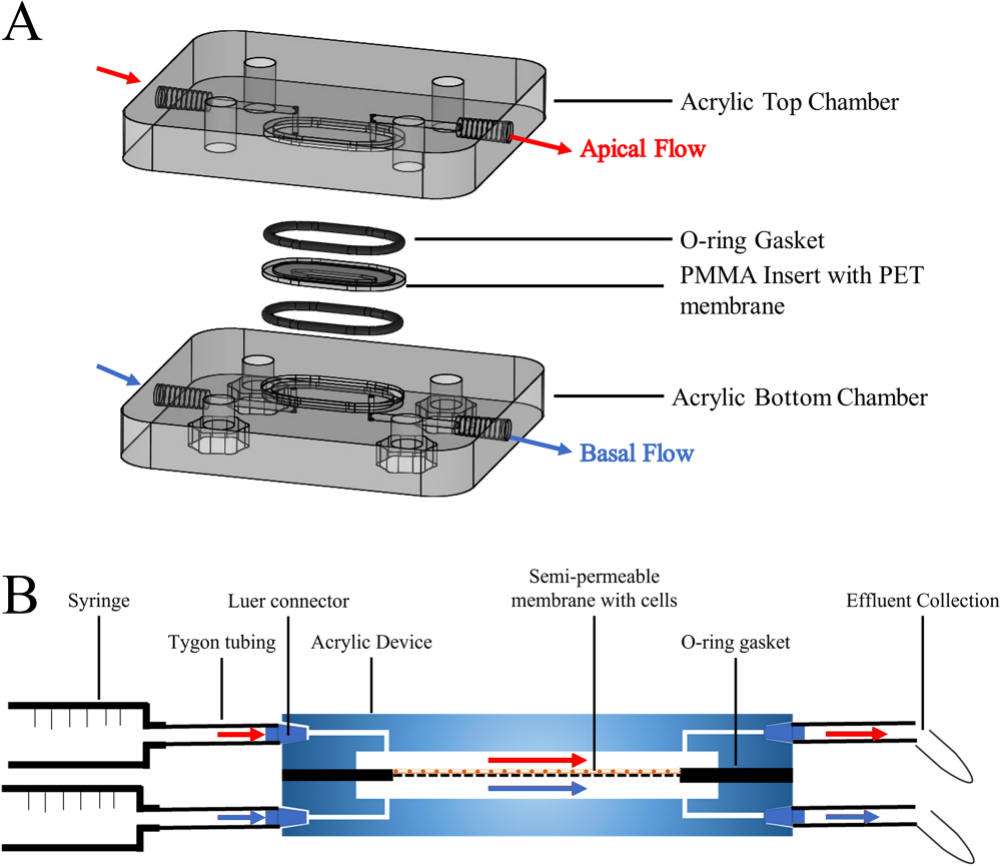
The dual-flow perfusion device. (a) Expanded schematic of the device, with acrylic top and bottom chambers, and a PMMA insert held in place between two O-rings (securing bolts excluded for clarity). (b) Schematic of device setup, with syringes connected to the apical and basal channels of the device. Medium was pumped through the device, connected with Tygon tubing and collected in polypropylene tubes. Detailed images of the device sections are shown in ESI (Figs. S1 and S2 in the supplementary material).

The device consists of two poly(methyl methacrylate), PMMA, (Kingston Plastics, Hull, UK) outer plates and a PMMA insert with a semi-permeable membrane designed to allow for the culture of cells. The two PMMA plates, both 60 × 70 mm^2^, were milled horizontally to produce 0.5 mm holes to which inlet and outlet silicone tubing could be attached via Luer elbow connectors. A central recess (24 × 10 mm^2^) was milled to house a removable insert, allowing direct flow across the chamber, and four holes with hexagonal recesses were made for the insertion of M6A2 stainless steel bolts to secure the unit [Fig. 1(a)]. A removable PMMA insert (24 × 10 mm^2^) containing a polyethylene terephthalate (PET) membrane, in a similar style to transwell inserts, was used for the culture of cells to enable ease of seeding [ESI, Fig. 1(a)]. The inserts were fabricated using a laser cutter (60W LS6840 laser, HPC Laser, Halifax, UK) to cut and engrave 1 mm thick PMMA sheets and to cut PET membranes (22 × 8 mm^2^, 8 *μ*m thickness, either 0.4 or 8 *μ*m pore size, Sabeau, Germany) to fit the carriers. Solvent bonding was used to adhere the PET membrane to the etched region of the PMMA insert and left to dry for 24 h. Prepared inserts were sterilized in 70% EtOH for at least 1 h before use. Although both 0.4 and 8 *μ*m pore size were investigated, the work presented below uses 0.4 *μ*m exclusively.

### B. Cell culture

The human colorectal adenocarcinoma cell lines, Caco-2 (HTB-37, ATCC, UK) and HT29-MTX-E12 (12040401, ECACC, UK) were maintained in Dulbecco's Modified Eagle's Medium (DMEM) with 10% (v/v) fetal bovine serum (FBS) and penicillin/streptomycin (100 IU ml^−1^ and 0.1 mg ml^−1^, respectively, all purchased from BioWhittaker, UK). The human umbilical vein endothelial cell line (HUVEC, PromoCell) was cultured in a complete low-serum endothelial cell growth medium (PromoCell), similarly supplemented with penicillin and streptomycin. Undifferentiated maintenance cultures of human neuroblastoma cell line SH-SY5Y (ECACC-94030304) were cultured in EMEM supplemented with 15% (v/v) FBS, 2 mM l-glutamine, 100 IU ml^−1^ penicillin, and 0.1 mg ml^−1^ streptomycin (all purchased from Sigma-Aldrich, Gillingham, UK). All cells were maintained in 75 cm^2^ tissue culture flasks (Sarstedt, Leicester, UK) at 37 °C in a humidified atmosphere containing 5% CO_2_.

For single cell line static or on-chip cultures, Caco2, HT29-MTX-E12, and HUVEC cells were seeded, 1 × 10^5^ cells in 100 *μ*l, onto the apical side of the semi-permeable PET membrane contained in the PMMA insert, and allowed to adhere for 72 h in a six-well plate. SH-SY5Y neuronal cells were differentiated according to a published method based on the sequential removal of serum from the medium (Shipley *et al.*, 2016). Cells were seeded on MaxGel Extracellular Matrix (Sigma) coated PMMA inserts at day 10 of the differentiation protocol and cultured for a further 8 days in differentiation media. The PMMA support was designed in house and printed using a Creator 2 3D printer using PLA filament (Farnell Element 14, Leeds, UK). The inserts with cells were subsequently placed into the perfusion device and secured with bolts. For the connected gut-brain model, Caco2, HT29-MTX-E12, or differentiated SH-SY5Y cells were cultured on the apical side of the semi-permeable membrane as above and HUVEC seeded onto the basal side of the membrane in the PLA support 72 h prior to placing into the connected perfusion devices. The inlets of the devices were connected to 20 ml sterile syringes with Tygon tubing. Continuous perfusion was carried out using a Harvard PhD 2000 syringe pump (Harvard Apparatus) at a flow of 2.94 *μ*l min^−1^ (Baldwin, 2020). The devices were maintained in a Covatutto 24 Eco incubator at 37 °C. Effluent was collected in 1.5 ml polypropylene tubes (Sarstedt) and stored at 4 °C until analysis.

### C. Assessment of membrane permeability

Membrane integrity was assessed using phenol red and phenol red free medium, flowed in the apical and basal channels, respectively, at 2.94 *μ*l min^−1^. Effluent was collected over the course of the experiment and analyzed for the presence of phenol red using a plate reader (Bio-TEK, Synergy HT) to measure absorbance at 558 nm.

Barrier permeability was assessed using Fluorescein isothiocyanate (FITC) dextran (Dawson *et al.*, 2016). In either a static or flow model, FITC dextran (0.5 mg ml^−1^, 10 kDa Dextran, Sigma) was added to, or flowed over the apical side of the membrane (2.94 *μ*l min^−1^). Samples were taken from the basal side of both models at set time points and the concentration of FITC-dextran determined using a fluorescent plate reader (480 nm excitation, 520 nm emission). A blank membrane was tested as a reference and to distinguish the effect of the cell monolayer compared with simple membrane permeability.

### D. Immunofluorescent staining of cells

Cells were stained for the presence of the tight junction protein ZO-1 (O'Rourke *et al.*, 2016). Cells were fixed with 4% (w/v) formaldehyde then quenched and permeabilized using NH_4_Cl/Triton-X100 solution (50 mM/0.2% v/v). Cells were washed twice with phosphate buffered saline pH 7.4 (PBS), blocked with 1% (w/v) bovine serum albumin (BSA) in PBS for 30 min, and incubated with primary antibody (ZO-1, Rabbit mAb, Biolabs, UK), diluted 1:1000 in a blocking buffer at room temperature for 1h. After incubation, cells were washed three times with the wash buffer (PBS with 0.05% Triton-X100) for 5 min each time on a rocking plate, and incubated at room temperature for 1h with the secondary antibody conjugated with AlexaFluor 488 (anti-rabbit IgG, Biolabs) diluted 1:500. Concanavalin A (ConA) conjugated with rhodamine (1:500 Vector labs, UK) was used for visualization of membranes, Alexa-488 conjugated Phalloidin (1:1000) was used for visualization of actin filaments (Abcam) and nuclei were visualized using Hoechst 33342 (ThermoFisher). Counterstains were incubated on the cells for 30 min before washing in PBS and the addition of the mounting medium (Vectorshield, Vector Labs, UK). Slides were imaged using a Zeiss LSM710 or LSM880 confocal microscope equipped with 63×/1.4 oil DIC objective and Zen black software (Zeiss).

### E. Periodic Acid-Schiff (PAS) staining of cells

Cells on membrane inserts were fixed in 4% (w/v) formaldehyde before transfer to 70% EtOH. Staining was carried out according to the manufacturer's instructions [Periodic Acid-Schiff (PAS) staining system, Sigma]. Cells on membranes were first immersed in the periodic acid Solution for 5 min before rinsing in three changes of distilled water. The membranes were then placed in Schiff's reagent for 15 min before rinsing in running tap water for 5 min. The membranes were counterstained in Harris Haematoxylin solution (Sigma) for 60 s. Counterstained membranes were rinsed in running tap water then dried by blotting on tissue and mounted using Hydromount™ mounting solution (National Diagnostics). Slides were dried overnight before imaging with an Olympus IX71 inverted fluorescence microscope equipped with a 40× objective and cellSens software (Olympus).

### F. Bt BEV isolation and labelling

BEVs were isolated and characterized as previously described (Fonseca *et al.*, 2022). Briefly, Bt (strain VPI 5482) was grown under anaerobic conditions at 37 °C in bacteroides defined medium (BDM), centrifuged at 6000 × *g* for 50 min at 4 °C and the supernatants filtered through polyethersulfone (PES) membranes (0.22 *μ*m pore-size, Sartorius) to remove debris and cells. Supernatants were concentrated by cross-flow ultrafiltration (100 kDa molecular weight cut-off, Vivaspin 50R, Sartorius), the retentate was rinsed once with 500 ml of PBS and concentrated to 1 ml. Further purification of BEVs was performed by the fractionation of the suspension by size-exclusion chromatography using qEV original 35 nm columns (Izon) according to the manufacturer's instructions. Fractions containing BEVs were finally combined and filter-sterilized through a 0.22 *μ*m PES membrane (Sartorius); suspensions were stored at 4 °C. Absence of viable microorganisms was confirmed by plate count and absence of lipopolysaccharide was confirmed by the Limulus Amebocyte Lysate (LAL) test (Sigma).

The size and concentration of the isolated Bt BEV suspension was determined by nanoparticle tracking analysis using the ZetaView PMX-220 TWIN instrument according to manufacturer's instructions (Particle Metrix). Aliquots of BEV suspensions were diluted 1000–20 000-fold in particle-free water for analysis. Size distribution video data were acquired using the following settings: temperature: 25 °C; frames: 60; duration: 2 s; cycles: 2; positions: 11; camera sensitivity: 80 and shutter value: 100. The ZetaView NTA software (version 8.05.12) was used with the following post-acquisition settings: minimum brightness: 20; max area: 2000; min area: 5 and trace length: 30.

For BEV translocation studies, Bt BEVs (1 × 10^11^ ml^−1^) were labeled with 5% (v/v) 1,1′-dioctadecyl-3,3,3′,3′-tetramethylindocarbocyanine perchlorate (DiD) Vybrant cell-labeling solution (Molecular Probes) by incubating at 37 °C for 30 min. The unbound dye was removed by washing with 3× PBS using centrifugal filters (100 kDa MWCO, Sartorius).

### G. BEV translocation assay

For the connected Caco-2 and differentiated SH-SY5Y (gut-brain) perfusion model, DiD-labeled Bt BEVs were added to the input medium (1 × 10^10^ ml^−1^) and flowed over the apical side of the membrane. Following removal from the device, cells on inserts were labelled with Alexa-488 conjugated Phalloidin for visualization of actin filaments (Abcam) and nuclei were visualized using Hoechst 33342 (ThermoFisher). Counterstains were incubated on the cells for 30 min before washing in PBS. Following excision from the inserts, cells on membranes were mounted on slides using Fluoromount-G mounting medium (ThermoFisher).

To visualize BEV translocation, cells from the gut-brain tissue perfusion model were imaged using a Zeiss LSM880 confocal microscope equipped with 63×/1.4 oil DIC objective and Zen black software (Zeiss). Fluorescence was recorded at 405 nm (blue, nucleus), 488 nm (green, phalloidin), and 647 nm (far-red, DiD-BEVs). Image analysis was performed using Image J/FIJI v1.52p.

### H. Statistical analysis

Data are presented as means ± standard deviation (SD).

## III. RESULTS

### A. Evaluation of flow in single and connected perfusion systems

**FIG. 2.**
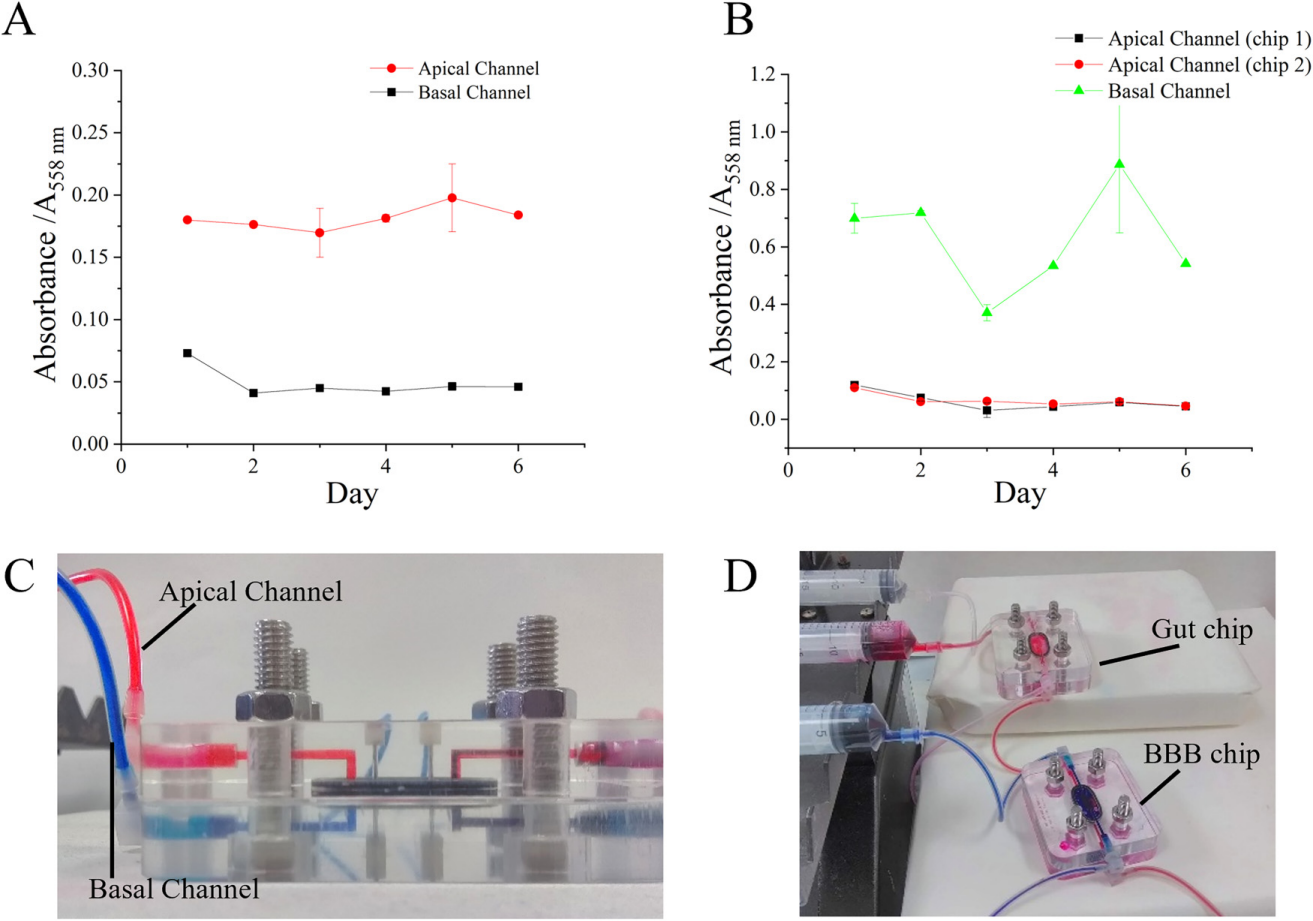
Assessment of flow within single and connected devices. (a) Absorbance of effluent collected from a single perfusion device over six days, measured using a plate reader. (b) Absorbance of effluent collected from two connected devices over six days; apical channel of the gut chip connected to the apical channel of the blood brain barrier (BBB) chip. Images of single (c) and connected (d) devices showing isolated flow are maintained in both channels throughout the experimental period. Data representative of five independent repeat experiments, error bars show standard deviation (SD).

Flow was assessed in both a single device and two devices connected through the basal channel by perfusing with two media streams, one containing phenol red and one phenol red free, both at a flow rate of 2.94 *μ*l min^−1^ for six days (chip conditions shown in ESI Table S1 in the supplementary material). Figure 2(a) shows that isolated flow can be maintained between the apical and basal channels of the device as shown by the difference in absorption between the phenol red and phenol red free channels, illustrated with the use of colored dyes [Figs. 2(c) and 2(d)] to visualize the flow. In Fig. 2(a), the phenol-red containing medium passed through the apical channel, in Fig. 2(b), the phenol-red containing medium went through the apical channel of chip 1 which is subsequently connected to the apical channel of chip 2. The experiment with two connected devices [Fig. 2(b)] showed that the flow could be maintained in individual channels when the devices were connected in series. Using the devices in series allowed modeling of multiple tissue barriers in a connected system. To further assess the device flow, sheer stress was calculated using the Navier–Stokes equation (Seymour *et al.*, 2020). At a flow rate of 2.94 *μ*l min^−1^, the cells were subjected to 4.0 × 10^−3^ dyn cm^−2^ shear stress.

Permeability was further investigated using FITC-labeled fluorescent dextran of 10 kDa (ESI Fig. S3 in the supplementary material) to assess both the flow and permeability of the membranes. While limited permeation of FITC-dextran was seen diffusing through the membrane, it was found that the pore size of the membrane (0.4 vs 8 *μ*m) was a more important factor in inhibiting transport across the membrane than cell confluency. However, 0.4 *μ*m was subsequently chosen over the 8 *μ*m membrane as it allowed for the support of cells without transfer of labeled dextran to the other side.

### B. Evaluation of colonic epithelial cell lines in the MPS

**FIG. 3.**
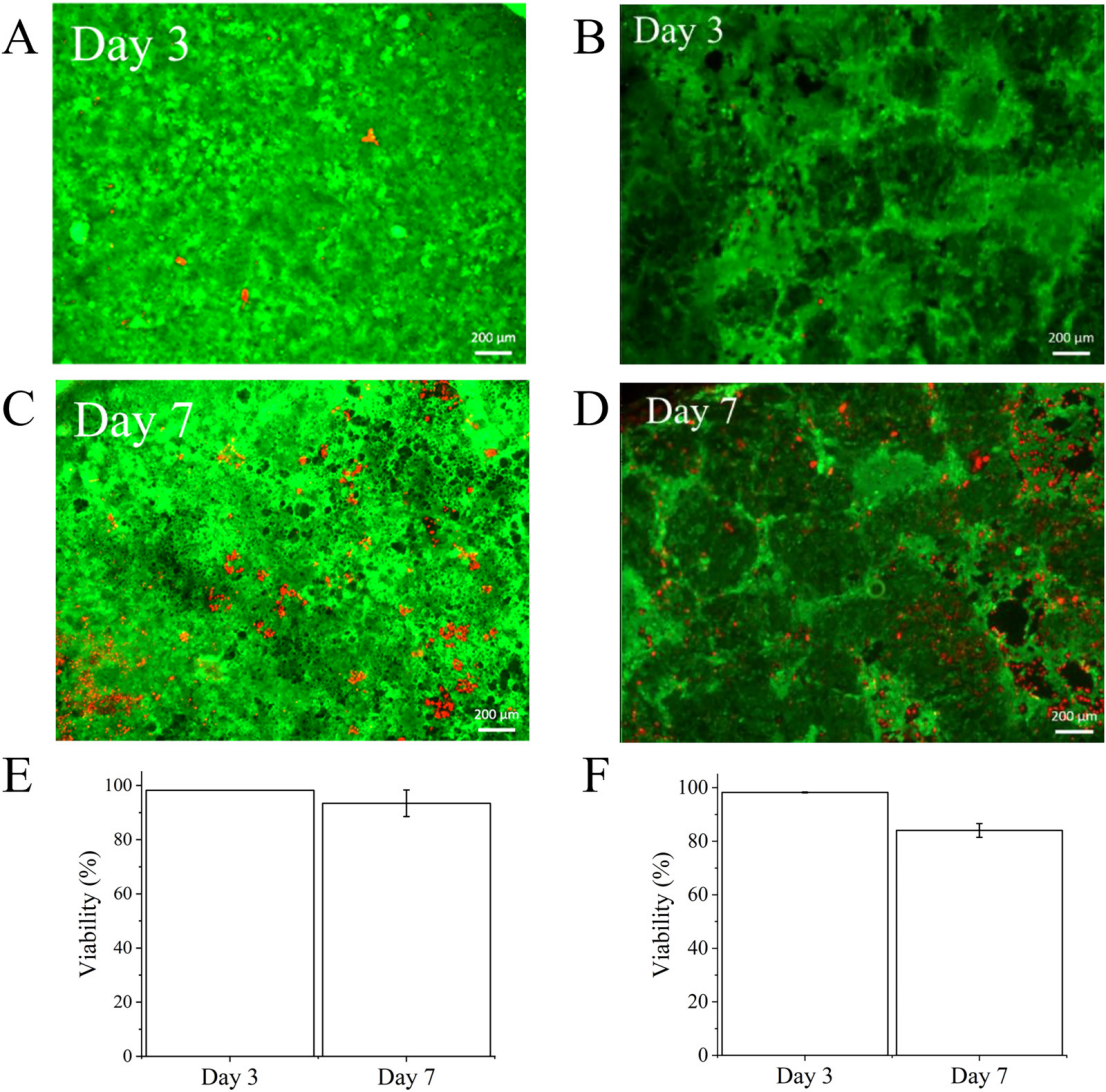
Viability of colonic epithelial cell lines assessed through FDA/PI staining. (a) Caco-2 cells or (b) HT29-MTX-E12 cells at 3 days on-chip. (c) Caco-2 cells or (d) HT29-MTX-E12 cells at 7 days on-chip. The bar charts depict the quantification of viability of Caco-2 cells (e) and HT29-MTX-E12 cells (f) maintained on-chip for 3 and 7 days using FDA/PI staining. Viable cells shown in green and dead cells in red. N = 3 devices, error bars show SD.

The single device was initially used to assess its ability to maintain apical cultures of two commonly used colonic cell lines. Caco-2 cell viability was maintained on-chip for up to 7 days under flow conditions [Figs. 3(a) and 3(c)] using an FDA/PI assay. The HT29-MTX-E12 cell line also retained viability for at least 7 days on the chips [Figs. 3(b) and 3(d)]. Cells reached 100% confluency by day 3 and remained confluent throughout the 7 day experiment. LDH assays carried out on effluent showed low levels of LDH were released which further confirmed cell viability (data not shown).

**FIG. 4.**
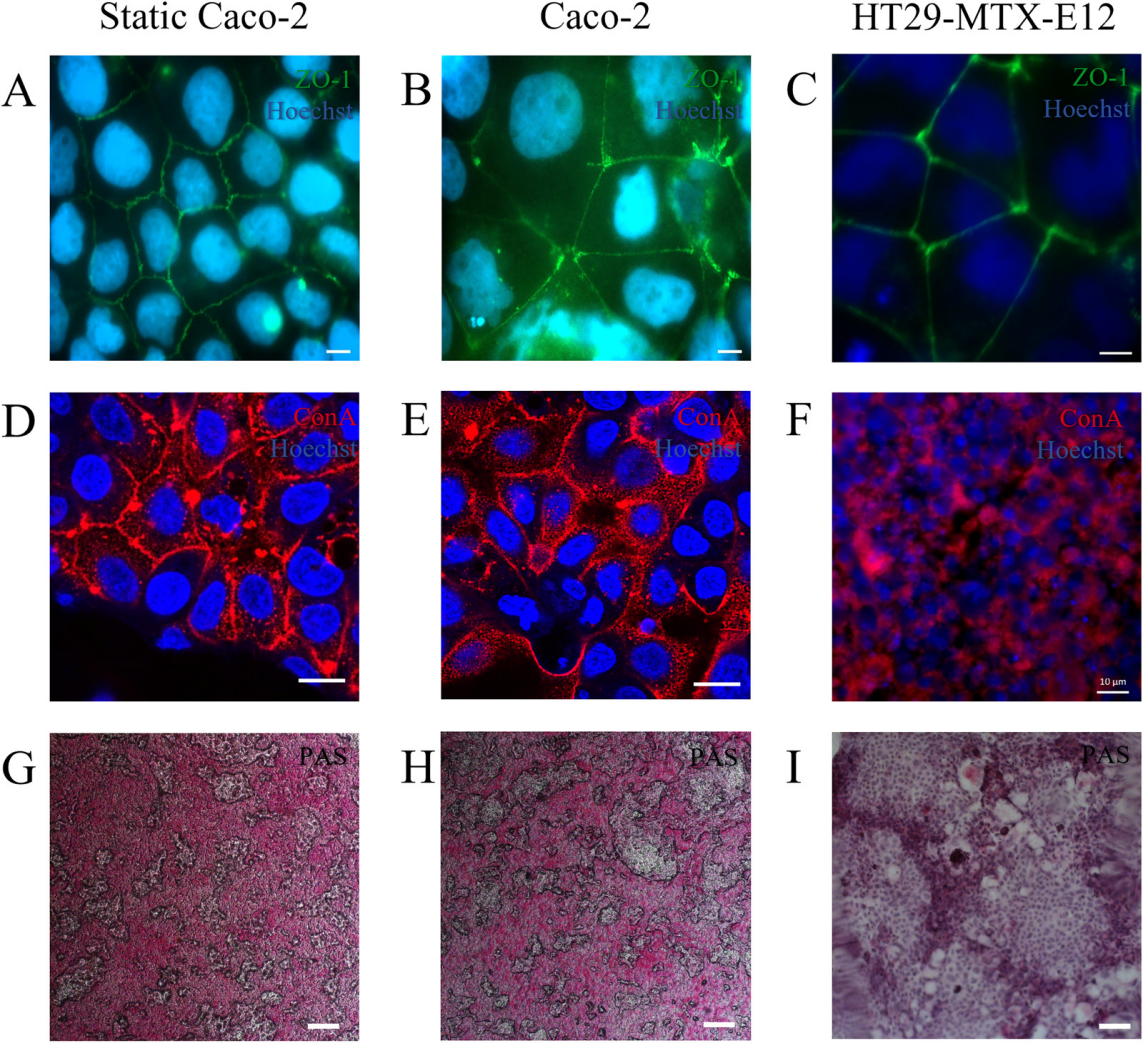
Immunofluorescent images of Caco-2 cells stained for ZO-1 under static (a) and on-chip conditions (b) and HT29-MTX-E12 cells in on-chip conditions (c) rhodamine-ConA labeled Caco-2 cells under static (d) and on-chip conditions (e) and HT29-MTX-E12 cell under flow conditions (f), n = 3. PAS stain was used to identify the presence of mucins (g)–(i). Images are representative of cells grown on two separate devices, n = 2. Scale bars 10 *μ*m.

The integrity of Caco-2 and HT-29-MTX-E12 cells grown under either static or flow conditions was examined by immunostaining with ConA to stain membrane glycoproteins highlighting cell walls, and an antibody specific for the cell membrane tight junction (TJ) protein ZO-1. By 72 h, Caco-2 cells formed a confluent monolayer under both conditions [Figs. 4(a), 4(b), 4(d), and 4(e)] with >90% of cells displaying ZO-1 staining consistent with a confluent monolayer (Anderson *et al.*, 1989). HT29-MTX-E12 cells had a similar appearance under flow conditions, generating a confluent monolayer of cells with uniform ConA and ZO-1 staining by 72 h on-chip [Figs. 4(c) and 4(f)]. Mucin secretion and PAS staining was seen in all culture conditions by day 5 of culture [Figs. 4(g)–4(i)].

### C. Evaluation of cell lines in the connected gut–brain MPS

**FIG. 5.**
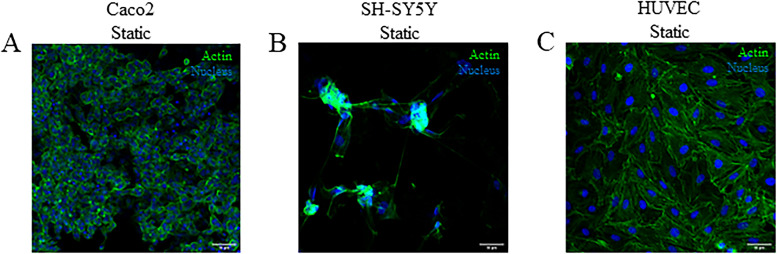
Cell line characterization in the gut-brain chip. Immunofluorescent images of Caco-2 cells (a), differentiated SH-SY5Y neuronal cells (b) and HUVEC endothelial cells (c) under static conditions for 24 h. Alexa-488 conjugated phalloidin stain for actin-microfilaments (green) and Hoechst nuclear stain (blue). Images are representative of >three independent experiments. Scale bars 50 *μ*m.

**FIG. 6.**
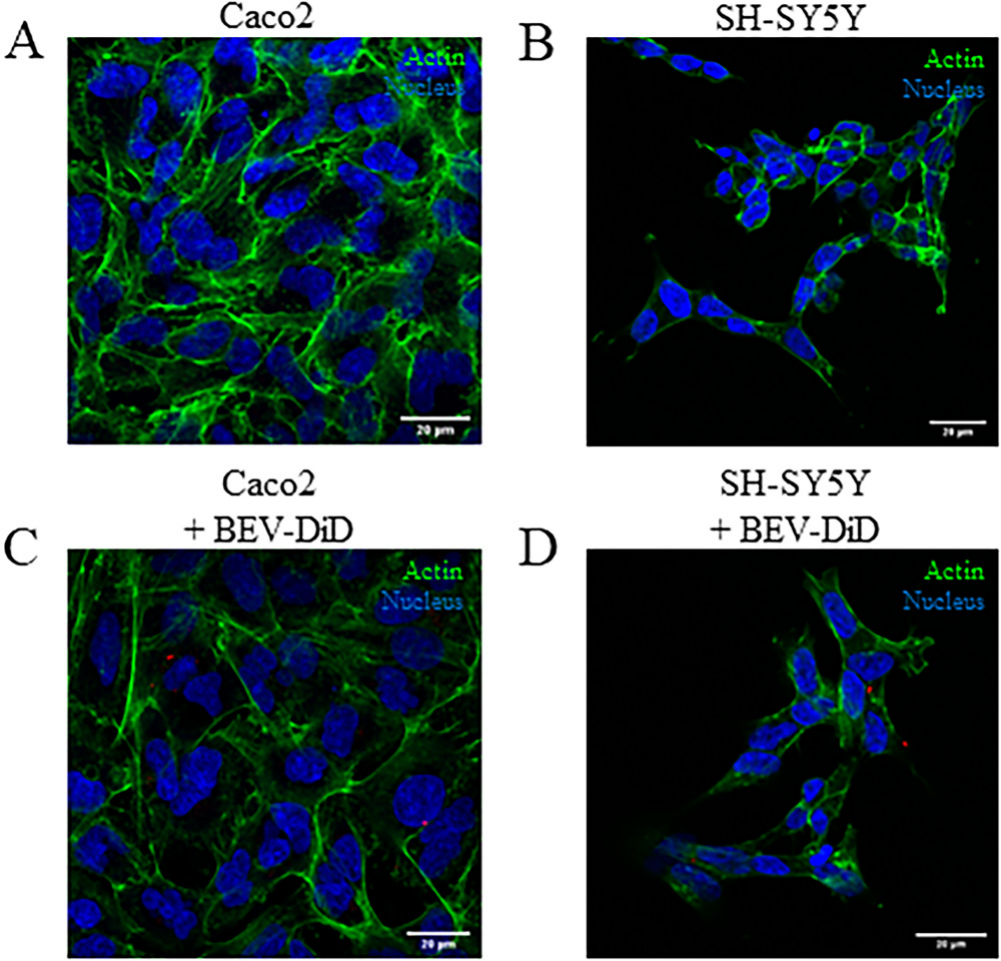
Visualization of Bt BEV translocation in connected gut-brain perfusion devices. Immunofluorescent images of cells cultured on gut-brain devices with Caco-2 (a) and differentiated SH-SY5Y cells (b) maintained under on-chip conditions for 24 h. Uptake and translocation of DiD-labeled bacterial extracellular vesicles (BEVs) between Caco-2 (c) and differentiated SH-SY5Y cells (d) in the gut- and brain-chips. Alexa-488 conjugated phalloidin stain for actin-microfilaments (green), Hoechst nuclear stain (blue) and DiD-labeled BEVs (red). Images are representative of two independent experiments. Scale bars 20 *μ*m.

Maintenance of Caco-2, differentiated SH-SY5Y, and HUVEC cell viability was first confirmed when cultured on the apical side of the semi-permeable membrane in PMMA inserts under static conditions [Figs. 5(a)–5(c)]. The cell lines were then cultured in connected gut-brain devices (ESI, Fig. 2). Caco2 or differentiated SH-SY5Y were cultured on the apical side of the semi-permeable membrane in the PMMA inserts and HUVEC endothelial cells were cultured on the corresponding basal side of the membrane in both devices. The gut–brain connected devices were maintained under flow conditions and morphology visually monitored, with no significant loss of viability observed in either device after 24 h [Figs. 6(a) and 6(b)].

### D. Connected devices as a gut–brain model: Trafficking and translocation of BEVs

Bt BEVs were used as a model component of the luminal gut microbiota to visualize translocation between the semi-permeable organ compartments of the connected gut-brain devices. At 24 h post inoculation of the apical medium, an intracellular punctate pattern of BEV uptake was observed in both the Caco-2 cells (gut-chip) and differentiated SH-SY5Y neuronal cells (brain-chip) cultured in the connected devices [Figs. 6(c) and 6(d)].

## IV. DISCUSSION

We describe here a new microphysiological system (MPS) platform consisting of connected perfusion devices that have been designed and developed to maintain a variety of cell lines for at least 7 days under flow conditions. Additionally, the devices were used to assess the acquisition and uptake of BEVs with established colorectal and differentiated neuronal cell monolayers which make up the majority of neuronal cells in the adult brain. Furthermore, the transport of BEVs across the gut-endothelial cell monolayers and uptake by brain neuronal cells has shown the suitability of the connected devices for modeling the translocation of substances between organ compartments.

Several microfluidic devices have been described previously that utilize flow in multiple channels connected by semi-permeable membranes. For example, Wright *et al.* (2023) used a PDMS based device to measure the permeability of mucin protein coated Caco-2 cells, with similar devices described by Choe *et al.* (2017) and Jeon *et al.* (2021) being used to create co-cultures of gut and liver cells as a model of the gut–liver axis. However, multi-component devices such as these often require complex, multi-staged, assembly and are limited to specific cell lines as they need to have similar doubling times and media compatibility. The use of disposable PMMA inserts in the devices described in the current work means that it is simple to seed multiple cell lines in static conditions before the introduction of flow, and include more complex models such as differentiated SH-SY5Y neuronal cells described here, then assemble connected devices when required. The integrity of the cell monolayers was demonstrated with cell viability being maintained at >85% at 7 days, when the experiments were stopped. Mucin secretion was detected in both static and flowing conditions, and although not quantified here previously this has been shown to be affected by shear stress (Navabi *et al.*, 2013). Current work is ongoing to determine how the cell monolayers cultured on the insert membranes behave over longer timepoints, and the applicability to a larger array of cell types and tissue or organ models.

*In vivo* shear stresses vary between 0.002 and 0.8 dyn cm^−2^ (Langerak *et al.,* 2020]. It is well known that changes in shear stress affect gene expression and cell function. A study by Delon *et al.* (2019) showed that increasing shear stress from 0.002 to 0.03 dyn cm^−2^ improved villi formation, increased F-actin production and tight junction formation in a cell monolayer. The calculated shear stress within the devices described here was 0.004 dyn cm^−2^, at the flow rate of 2.94 *μ*l min^−1^ which is relatively low in comparison with the PDMS device described by Kim *et al.* (2012), that reported a sheer stress of 0.02 dyn cm^−2^, although both fall within the physiological range. Bein *et al.* (2018) demonstrated the mechanical effects of different flows on the physiological response of a gut model *in vitro*. Shear stress can also be modified by the use of hydrogels to provide a type of ECM that supports the cell line and has been used to provide a framework for more complex 3D structures, as in the device described by Kim *et al.* (2021), which we did not investigate here, although it could be explored later to further improve the physiological relevance of the MPS device.

An important facet of most microfluidic devices is the ability to sample effluent repeatedly for analyte detection. The new devices described here allow this to be done as required, offering flexibility, as well as the easy removal of the cell monolayer for staining or subsequent analysis. A noted limitation of organ-on-a-chip devices is the low levels of biomarkers obtained from the effluent which minimizes the analysis able to be carried out. This has also been reported by groups such as Jeon *et al.* (2020) who have developed ways of increasing sensitivity through recycling of the culture. This has been done using reservoirs to contain small amounts of medium that are perfused repeatedly through the microfluidic device using a rocking mechanism. We have not found sensitivity to be an issue with cells and tissue maintained in other devices we have developed; preliminary investigations confirmed that IL-6 was reliably detected (data not shown). Similar devices used by Riley *et al.* (2021) also successfully identified a panel of factors in effluent using a Proteome Profiler (Biotechne, Abingdon, Oxford). If biomarker release was determined to be too low it is possible to concentrate the effluent, however there are limitations to this as additives to cell medium such as serum can interfere with any subsequent assays once concentrated.

A further additional advantage of the cell-line based MPS developed here is the ease and flexibility of setup. Specifically, inserts can carry different cell layers and cell culture can be developed off-chip and then introduced to on-chip flow conditions, overcoming problems with initial growth and establishment. Additionally multiple devices can be linked with each possessing a unique cell type, with effluents being sampled as appropriate. The ability to use well-characterized cell lines means that the devices can easily be adopted by other research groups, without requiring the often difficult and costly processes concerned with involving surgical collaborators providing fresh human tissue material. Finally, the devices have been designed to a scale that makes them suitable for use in Biosafety Level 3 and 4 (BSL3 and 4) environments where a worker's manual dexterity is compromised.

We have previously shown that Bt BEVs can be transported across the intestinal epithelium *in vivo* to reach distant tissues (Jones *et al.*, 2020). Due to their nano-size, BEVs can directly translocate across multiple barriers including the intestinal epithelial cell layer, the blood endothelial layer and ultimately the BBB to reach the brain (Modasia *et al.*, 2023). This has been studied within microfluidic devices by other groups, such as a PDMS device described by Kim *et al.* (2021) where they culture multiple cell lines within a single device. This work described the transport of exosomes between the gut and BBB and it was noted that the addition of flow could improve uptake of exosomes to the BBB, however this was only seen within the BBB part of the device (Kim *et al.*, 2021). The gut-brain MPS we have developed incorporates flow and showed that BEVs could be transported not only across the intestinal epithelium of the GIT-chip but can also be transported to a secondary brain-chip device, where they interact with, and are endocytosed by the neuronal cells.

## V. CONCLUSION

We describe a new and flexible tissue perfusion platform that can be joined in series to study cell and tissue processes, specializing in transport across epithelial and endothelial membranes. The device is fabricated from PMMA, which can be re-used multiple times, with an inexpensive carrier that allows simple loading and removal of cell cultures maintained under flow conditions. Here, we have exemplified the platform's attributes by successfully demonstrating the transport of Bt BEVs across epithelial and endothelial layers in dual gut-brain devices. We are currently using the platform to study the transmission of SARS-CoV-2 between the dual connected devices to develop a model of long-covid, exploiting the scale of the device that make it highly amenable for use in BSL3 and 4 facilities.

## SUPPLEMENTARY MATERIAL

See the supplementary material for further details of the device fabrication, flow conditions, and shear stress.

## Data Availability

The data that support the findings of this study are available from the corresponding author upon reasonable request.
